# Multienvironment genomic prediction in tetraploid potato

**DOI:** 10.1093/g3journal/jkae011

**Published:** 2024-01-18

**Authors:** Stefan Wilson, Chaozhi Zheng, Chris Maliepaard, Han A Mulder, Richard G F Visser, Fred van Eeuwijk

**Affiliations:** Biometris, Wageningen University & Research Centre, Wageningen, PB 6708, The Netherlands; Biometris, Wageningen University & Research Centre, Wageningen, PB 6708, The Netherlands; Plant Breeding, Wageningen University and Research, Wageningen, PB 6708, The Netherlands; Wageningen University and Research Animal Breeding and Genomics, Wageningen, AH 6700, The Netherlands; Plant Breeding, Wageningen University and Research, Wageningen, PB 6708, The Netherlands; Biometris, Wageningen University & Research Centre, Wageningen, PB 6708, The Netherlands

**Keywords:** genomic prediction, GenPred, shared data resource, multienvironment trials, genotype by environment interaction, tetraploid potato

## Abstract

Multienvironment genomic prediction was applied to tetraploid potato using 147 potato varieties, tested for 2 years, in 3 locations representative of 3 distinct regions in Europe. Different prediction scenarios were investigated to help breeders predict genotypic performance in the regions from one year to the next, for genotypes that were tested this year (scenario 1), as well as new genotypes (scenario 3). In scenario 2, we predicted new genotypes for any one of the 6 trials, using all the information that is available. The choice of prediction model required assessment of the variance–covariance matrix in a mixed model that takes into account heterogeneity of genetic variances and correlations. This was done for each analyzed trait (tuber weight, tuber length, and dry matter) where examples of both limited and higher degrees of heterogeneity was observed. This explains why dry matter did not need complex multienvironment modeling to combine environments and increase prediction ability, while prediction in tuber weight, improved only when models were flexible enough to capture the heterogeneous variances and covariances between environments. We also found that the prediction abilities in a target trial condition decreased, if trials with a low genetic correlation to the target were included when training the model. Genomic prediction in tetraploid potato can work once there is clarity about the prediction scenario, a suitable training set is created, and a multienvironment prediction model is chosen based on the patterns of G×E indicated by the genetic variances and covariances.

## Teaser

Wilson et al. combined information across trials and applied genomic prediction to tetraploid potato, while evaluating different modelling strategies and application scenarios.

## Introduction

Biologically speaking, the traits of an organism are a result of the organism’s genetic composition, external environmental stimuli and the interactions between these 2 over time. For prediction purposes, it is important to identify genotype by environment interactions (G×E), i.e. differences between the genotypic effects of a certain trait that depend on the environmental conditions (Malosetti *et al.* 2016; van Eeuwijk FA *et al.* 2016). Understanding G×E is especially important in plant breeding, as breeders may be interested in developing varieties for a target population of environments (Chenu 2015; Bustos-Korts *et al.* 2021), or in later stages of the breeding program, identifying those varieties which are stable across different sets of environmental conditions.

Cultivated potato (*Solanum tuberosum* L.), is one of the most consumed food crops and is grown in many environments across the world (Birch *et al.* 2012; Zaheer and Akhtar 2016). Studies analyzing the stability/adaptability of potato have been conducted previously (De Jong *et al.* 1981; Baril *et al.* 1995; Cotes *et al.* 2002; Affleck *et al.* 2008); however, these studies did not incorporate the wealth of genotypic information available today.

Current genotyping technology allows for significant coverage of the genome, resulting in thousands (up to millions) of markers. Coupled with the advancement of computing power and statistical methodologies, marker-assisted selection has become more common in plant breeding programs. Genomic prediction (GP) is a type of marker-assisted selection, where known phenotypes are regressed against marker profiles to estimate marker effects and/or breeding values (Bernardo 1996; Whittaker *et al.* 2000; Meuwissen *et al.* 2001), which are then used to predict the phenotypes of new material. Even though GP was developed over 2 decades ago, it has only been recently applied to potato (Slater *et al.* 2016; Sverrisdóttir *et al.* 2017; Enciso-Rodriguez *et al.* 2018; Endelman *et al.* 2018; Stich and Van Inghelandt 2018; Wilson *et al.* 2021; Ortiz *et al.* 2022) and so far only 1 paper has included G×E (Ortiz *et al.* 2022), and 2 others have investigated the interaction between genotype and year (Enciso-Rodriguez *et al.* 2018; Endelman *et al.* 2018). The analysis of Ortiz *et al.* (2022) showed that for tuber weight in particular the inclusion of G×E in multienvironment prediction models resulted in more accurate predictions than models that did not include the G×E interaction. In that study, varieties were put in classes depending on their tuber size and the increase in prediction ability gained from combining environments, varied depending on the class for which predictions were being made. In this study, we will also analyze tuber weight, but include more traits and multienvironment GP models to uncover any trait-specific considerations that should be made when performing multienvironment prediction.

The extent to which G×E interaction can be beneficial to GP of a given trait is largely dependent on the genetic correlation between environments (van Eeuwijk F *et al.* 2008; Malosetti *et al.* 2013). In scenarios where there is a high genotypic correlation between environments, meaning that varieties perform similarly in each environment and G×E interactions are small, simply using a model without G×E and only a genotypic main effect will increase prediction ability. In situations with substantially low correlations between environments, the combining of information will not be beneficial, regardless of the model. When there is moderate correlation between environments, we can benefit in combining information across them by adding a G×E term to the genotype main-effect multienvironment model (Burgueño *et al.* 2012; Windhausen *et al.* 2012).

There are various scenarios where multienvironment GP is useful, and in this study, we consider 3 specific scenarios. Scenario 1: breeders want to predict for a known region in an upcoming year, the performance of current genotypes (Malosetti *et al.* 2016; Enciso-Rodriguez *et al.* 2018; Endelman *et al.* 2018). Scenario 2: predicting the performance of new genotypes for a particular set of conditions given all the information collected so far on tested genotypes (Burgueño *et al.* 2012; Windhausen *et al.* 2012). Scenario 3: a combination of scenarios 1 and 2, where the performance of new genotypes for a region in the next year is predicted, using all current training information (Malosetti *et al.* 2016; Enciso-Rodriguez *et al.* 2018; Endelman *et al.* 2018). How well this can be done largely depends on how closely environments are related, which can be inferred by the degree of similarity between the ranking of genotypes in the different environments. The potential for predicting the new untested genotypes depends on how related they are to tested genotypes.

Strategies for modeling G×E interactions have been studied and reviewed considerably (van Eeuwijk F *et al.* 2008; van Eeuwijk FA *et al.* 2016). The choice of model largely depends on the genetic architecture of the trait, the depth of environmental information available and the number of parameters to be estimated. These parametrization considerations must be made when incorporating G×E interactions into a GP mixed model, as the G×E covariance matrix can become quite large and complex. Traits that exhibit simple G×E patterns can be modeled with covariance structures that assume homogeneous relationships between environments and require less parameters to be estimated. On the other hand, for more complex G×E interactions, we use covariance structures that capture a specific relation between environments, and therefore more parameters need to be estimated (Burgueño *et al.* 2012; Malosetti *et al.* 2013). Model parameters can be estimated via maximum likelihood or Bayesian resampling. Bayesian GP models have grown in popularity and its extension to G×E has also seen increased interest (Cuevas *et al.* 2017; Montesinos-Lopez *et al.* 2018); however, maximum likelihood is far more time efficient (Ferrão *et al.* 2019), and the evaluation of different model parametrizations can be done by comparing log-likelihoods.

Using high-density molecular markers, and phenotyping information collected from 3 regions across 2 years, we will perform multienvironment GP to tetraploid potato across the aforementioned breeding scenarios. For 3 traits (dry matter, tuber weight, and tuber length) and 6 trials, the magnitude of G×E will be quantified and used to assess the prediction abilities of various multienvironment models and G×E covariance structures. The aim is to get an impression of G×E in tetraploid potato within and across Europe, while investigating the suitability of the different modeling techniques for the different breeding scenarios and G×E patterns. In addition, we will briefly look at how multienvironment prediction is affected by the relatedness of environments included in the model, by investigating the genetic correlations between environments.

## Materials and methods

### Phenotype and genotype information

This study looked at a diversity panel of 147 tetraploid potato genotypes that mainly consisted of commercial varieties; however, recent Dutch breeding material was included. Genotypes were classified into 7 distinct market classes: ancient, chip processing, French fry processing, fresh consumption, starch, cooking, starch and other. The population structure of this panel was explored in a recent study, and showed very little separation between subpopulations (Wilson *et al.* 2021). Wright $F_{ST}$ statistics between market classes were close to zero and in an analysis of molecular variance, population classifications contributed only 6.7% of the total molecular variation (Wilson *et al.* 2021).

In 2017 and 2018, field trials were conducted in Głubczyce in Poland (N $50^{\circ }13^{\prime }29.2^{\prime \prime }$, E $17^{\circ }48^{\prime }40.1^{\prime \prime }$), Ecija in Spain (N $37^{\circ }32^{\prime }51.1^{\prime \prime }$, W $5^{\circ }12^{\prime }30.2^{\prime \prime }$), and Emmeloord The Netherlands (N $52^{\circ }40^{\prime }06.2^{\prime \prime }$, E $5^{\circ }42^{\prime }00.2^{\prime \prime }$). At all locations, locally optimized management practices were followed concerning planting date, fertilization, irrigation, and pest control. Plots consisted of 8 plants with 2 ridges and 4 plants per ridge, spaced in such a way that there was 0.75 m between ridges and 0.33 m between plants in Spain and the Netherlands. In Poland, there was 0.9 m separation between ridges and 0.29 m between plants. In each trial, which was a location by year combination, a row–column resolvable design was implemented with 2 complete blocks. Latinization over rows and columns was used to disperse varieties across the field. Randomization was performed using the package DiGGer (Coombes 2009) executed with the software R (R Core Team 2019).

In this study, we will focus on 3 traits: tuber weight per plot (kilograms), mean tuber length (millimetres), and dry matter content (percentage). For each trial, a mixed model that corrected for block effects and random row and column effects was used to extract the adjusted phenotypic means. The resulting best linear unbiased estimates (BLUEs) were used as the dependent variable for GP models. Using this same mixed model, with all terms remaining the same except genotype effects are changed from fixed to random, genetic variances were extracted and used to calculate trait heritability for each trial. The genetic architectures of the traits in this diversity panel were explored separately in a previous study, and the results showed that additive markers were able to explain 13, 42, and 67 of the phenotypic variance in tuber weight, tuber length, and dry matter, respectively (Wilson *et al.* 2021).

Genotyping by sequencing, aligning with the reference genome, and filtering resulted in a marker matrix of 39,000 single nucleotide polymorphism markers, across the 147 individuals. For more details on the genotyping process, please see Wilson *et al.* (2021). Each element of the marker matrix gives the allele dosage, i.e. the discrete count of alternative alleles (0, 1, 2, 3, 4). When these counts are entered in a design or relationship matrix and a single parameter is estimated to quantify the dependence of the phenotype on the allele count, then this implies that marker effects are additive.

### Estimating G×E and statistical models

For GP, we use a statistical mixed model with random genomic effects. To extend this to a multienvironmental model, an environmental main effect is added, and can be further extended by including G×E interaction effects: random genetic effects specific to each environment. This requires a covariance structure with genetic correlations for all environment combinations, estimated via maximum likelihood. Simplified genetic correlation structures can be used to restrict the number of parameters to be estimated, however may oversimplify the G×E pattern of a given trait. Traits with little G×E do not require complex models for the genetic correlations between environments, whereas traits that have complex G×E patterns may need more flexibility. Using restricted maximum likelihood via ASREML (Butler 2009), various models were compared for all traits, and evaluated according to prediction ability and log-likelihood.

It should be noted that in the description of the models, we use the word environment, however depending on the prediction scenario which will be discussed later, the environments can refer to the trials, or can refer the 3 regions.

- *Single environment G-BLUP model (SE)*
(1) $$y_{i}=\mu +g_{i}+\epsilon_{i},$$
  where $y_{i}$ is the observation of genotype *i*, *μ* is the overall mean for the environment, $g_{i}$ is the random effect of genotype *i*, with distribution: $g_{i}∼N(0,G\sigma_{g}^{2})$. *G* is the additive genomic relationship matrix (from allele dosages) based on the work of VanRaden (2008) and extended by Ashraf *et al.* (2016). The term $\epsilon_{i}$, with distribution $\epsilon_{i}∼N(0,\sigma_{\epsilon }^{2})$, and variance $\sigma_{\epsilon }^{2}$, is a random residual that contains genetic effects that are not modeled by the $g_{i}$ term as well as nongenetic effects, like the plot error. This model is used to make predictions within a single environment.
- *Multienvironment G-BLUP models*
(2) $$y_{ij}=\mu_{j}+g_{ij}+\epsilon_{ij},$$
  where $y_{ij}$ is the BLUE of genotype *i* in environment *j*, $\mu_{j}$ is the mean of environment *j*. The residual for genotype *i* in environment *j* is denoted by $\epsilon_{ij}$, therefore modeling heterogeneous residuals across environments with a multivariate normal distribution $\epsilon_{ij}∼MVR(0,R)$, where *R* is a diagonal matrix of environment specific residual variances ($\sigma_{j}^{2}$). The term $g_{ij}$ is the random effect of genotype *i* within environment *j*. As a result $g_{ij}$ also has a multivariate normal distribution, such that $g_{ij}∼MVR(0,\Sigma )$, where $\Sigma =G⊗\Sigma_{E}$. The term *G* represents the genomic relationship matrix as seen in the previous single environment model, and the term $\Sigma_{E}$ represents the genetic covariance matrix between environments. Four parametrizations of this matrix were compared in this study.

1. Main effects (ME): The covariance matrix between environments is simply a matrix of all 1’s. If, for example, we were to consider 3 environments, our covariance matrix will look like:
  $$\Sigma_{E}=\left[\begin{array}{ccc} 1 & 1 & 1\\ 1 & 1 & 1\\ 1 & 1 & 1 \end{array}\right].$$
  As previously mentioned, variance of genotype effects for the multienvironment model is calculated as $\Sigma =G⊗\Sigma_{E}$. Therefore, for the main environmental effects model, the genetic variance ($\sigma_{g}^{2}$) is always multiplied by 1, and therefore remains the same regardless of environment. This homogeneous variance across environments means there is no G×E interaction. Additionally, the correlation between environments is 1, therefore no G×E. The only term that differentiates environments is the main environmental effect ($\mu_{j}$).
2. Compound symmetry (CS): In compound symmetry, each environment has the same variance, and the covariances between pairs of environments are also identical. This is the simplest G×E parametrization in this study, and assumes a uniform relationship between and across environments.
  $$\Sigma_{E}=\left[\begin{array}{ccc} \sigma_{1}^{2} & \sigma_{2}^{2} & \sigma_{2}^{2}\\ \sigma_{2}^{2} & \sigma_{1}^{2} & \sigma_{2}^{2}\\ \sigma_{2}^{2} & \sigma_{2}^{2} & \sigma_{1}^{2} \end{array}\right].$$
  The parameter $\sigma_{1}^{2}$ is the genetic main-effect variance plus the variance for G×E and is homogeneous across environments as in the previous model (ME). However, this model includes the term, $\sigma_{2}^{2}$ which is the covariance between environments. The correlation between environments can be calculated as $\frac{\sigma_{2}^{2}}{\sigma_{1}^{2}}$.
3. Uniform heterogeneity (UN.het): The full name of this model is “uniform correlation—heterogeneous variances” and as the name indicates, each environment has its own unique variance, so the G×E expresses itself as heterogeneity of variance. Like the compound symmetry model, the correlation between environments is constant; however, the covariances between environments are not.
  $$\Sigma_{E}=\left[\begin{array}{ccc} \sigma_{1}^{2} & \rho \sigma_{1}\sigma_{2} & \rho \sigma_{1}\sigma_{3}\\ \rho \sigma_{1}\sigma_{2} & \sigma_{2}^{2} & \rho \sigma_{2}\sigma_{3}\\ \rho \sigma_{1}\sigma_{3} & \rho \sigma_{2}\sigma_{3} & \sigma_{3}^{2} \end{array}\right].$$
  The parameter $\sigma_{1}^{2}$ is the genetic main-effect variance plus the variance for G×E in environment 1. The covariance between environments 1 and 2 is given by $\rho \sigma_{1}\sigma_{2}$ and is therefore proportional to the individual variances, multiplied by the constant correlation (*ρ*).
4. Unstructured (US): Genetic variances are unique for environments, and genetic correlations are unique for pairs of environments. This is the most complex G×E model used in this study, requiring the highest number of parameters to be estimated.
  $$\Sigma_{E}=\left[\begin{array}{ccc} \sigma_{1}^{2} & \sigma_{12} & \sigma_{13}\\ \sigma_{12} & \sigma_{2}^{2} & \sigma_{23}\\ \sigma_{13} & \sigma_{23} & \sigma_{3}^{2} \end{array}\right].$$
  The genetic correlations between environments were estimated using this parametrization. For example, the genetic correlation between environments 1 and 2 are calculated as $\rho_{12}=\frac{\sigma_{12}}{\sigma_{1}\sigma_{2}}$, where $\sigma_{1}^{2}$ and $\sigma_{2}^{2}$ represent the genetic main-effect variance plus the variance for G×E in environments 1 and 2, respectively, and $\sigma_{12}$ is the covariance between these environments.

The unstructured model contains genetic correlations which allow for the inference of G×E patterns for each trait; high genetic correlations between environments mean lower G×E interaction, and vice versa. A genotype plus genotype by environment (GGE) biplot was used to quickly visually inspect the variance–covariance structure of the observations across environments ($y_{ij}$), in the hope to see where the heterogeneity of variance and correlation is located (Bernal *et al.* 2013). For each trait, the length of each arrow represents the genetic variance within an environment, while the cosine of the angle between arrows represents the genetic correlation between environments (Yan *et al.* 2000).

### Prediction scenarios

The performance of the aforementioned models were evaluated in various applications or scenarios where multienvironment predictions may be useful.

#### Scenario 1: Predicting tested genotypes for future years

Otherwise known as forecasting, we want to predict future performance of tested genotypes in a particular region, using past phenotypic information from that region. As it pertains to this study, we will try to predict the genotypic performance of cultivars at a region in 2018, using the phenotypic information of 2017 and vice versa.

**Fig. 1.**
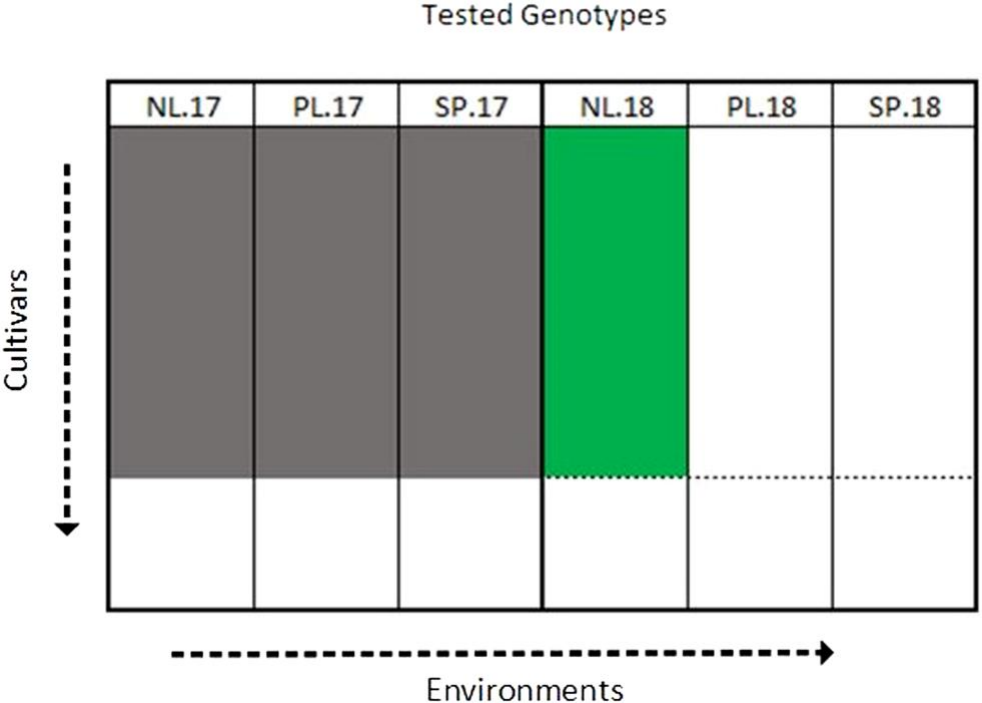
Illustration of scenario 1 for predicting in Netherlands 2018 (NL.2018) using 2017 data. The gray regions represent the training set and the green region represents the prediction set.

Using 105 individuals in the training set, we wish to predict the performance of cultivars in Netherlands 2018 (for example) using data from Netherlands 2017 only vs combining the data from all 2017 trials. Prediction ability is the correlation between the predicted genotypic effects and their observed phenotypic values, averaged over 100 repetitions, i.e. 100 divisions of training and test set. Figure 1 illustrates the case where predictions are to be made in Netherlands 2018 from all 2017 data. This was performed in all three 2018 regions, and repeated in the reverse direction where predictions are made in all 2017 regions, using the 2018 data to train the model.

#### Scenario 2: Predicting untested genotypes in known conditions

**Fig. 2.**
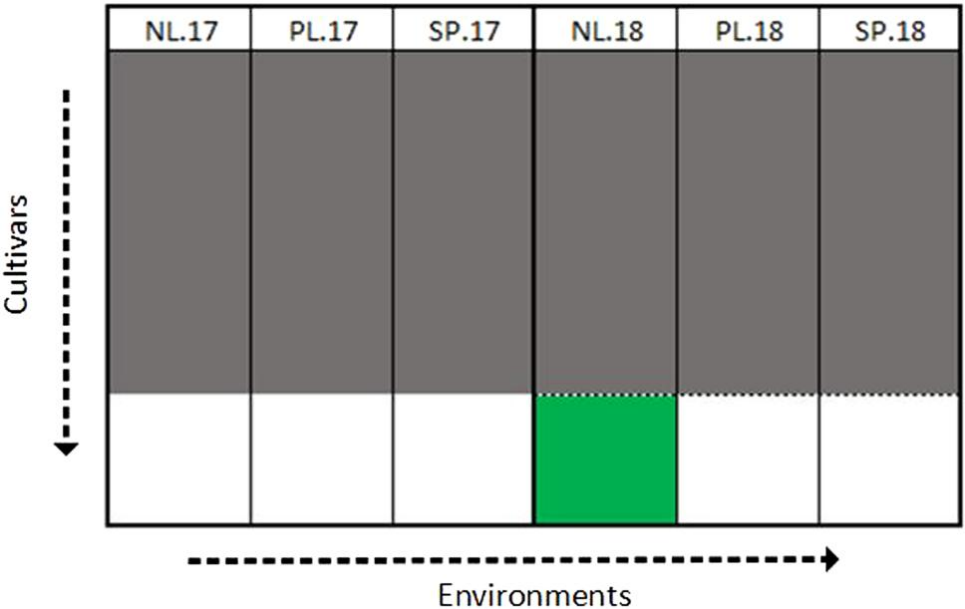
Illustration of scenario 2 for predicting in Netherlands 2018 (NL.18). The gray regions represent the training set and the green region represents the predicted set. This was repeated with the predicted set in each of the other 5 trials. The predicted individuals are excluded from the training set making these untested genotypes.

In this scenario, we want to predict new genotypes from all information available, for a particular set of conditions (a year within a region). For this study, we have 6 different trials or trial conditions (NL17, PL17, SP17, NL18, PL18, SP18). For each trial, 105 cultivars were chosen from each trial to train the model and predict the held out individuals, and repeated 100 times. Prediction ability is the correlation between the predicted genotypic effects and their observed phenotypic values, averaged over the 100 repetitions. In Fig. 2, we see an example where we make predictions of new genotypes in Netherlands 2018, with a model that combines information over all 6 trials. As mentioned before, various multienvironment models are compared, along with the single environment model.

#### Scenario 3: Predicting untested genotypes for future years

Like scenario 1, we wish to make predictions in a particular region, however these are for genotypes not included in the training set. This is a more ambitious application of multienvironment prediction because for the genotypes to be predicted there is no previous information (unlike scenario 1), and limited information on the particular environmental conditions (unlike scenario 2).

**Fig. 3.**
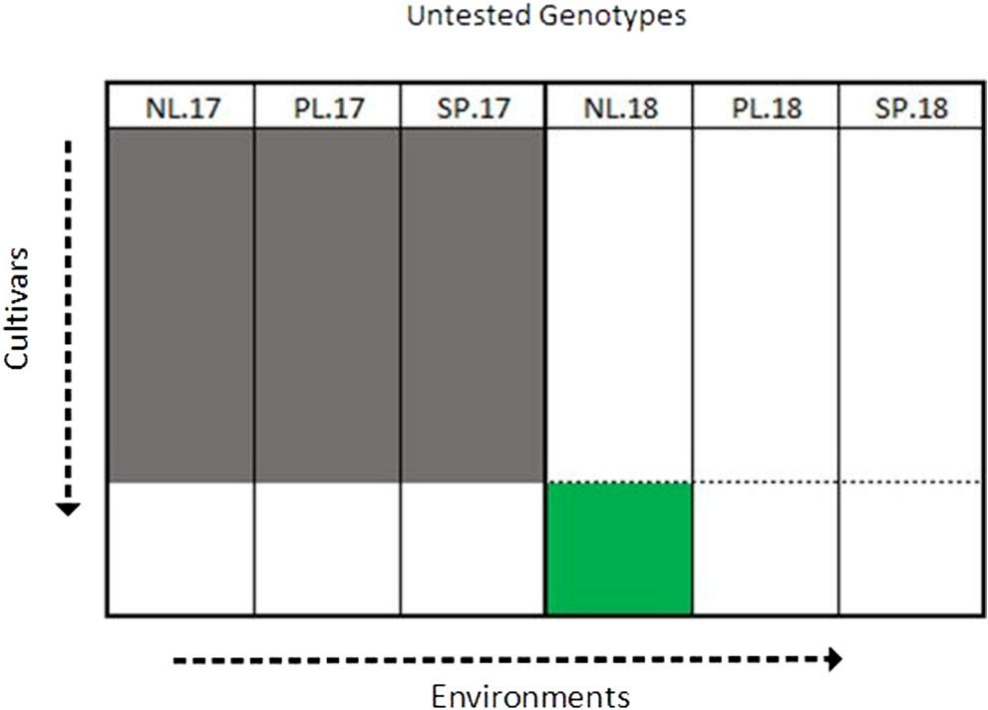
Illustration of scenario 3 for predicting in Netherlands 2018 (NL.2018) using 2017 data. The gray regions represent the training set and the green region represents the prediction set.

In this scenario, we want to predict the performance of untested genotypes in Netherlands 2018 using data from Netherlands 2017 only vs combining the data from all 2017 trials as illustrated in Fig. 3. Prediction ability is the correlation between the predicted genotypic effects and their observed phenotypic values, averaged over 100 repetitions. Similar to scenario 1, this was performed in all three 2018 locations, and repeated in the reverse direction where predictions are made in all 2017 locations, using the 2018 data to train the model.

## Results

###  

#### Trait heritability

Broad-sense heritability was estimated during phenotypic analysis as the proportion of phenotypic variation that can be attributed to genetic variation (Table 1).

**Table 1.** Heritability estimates of each trait at each field trial.

	Tuber weight	Tuber length	Dry matter
NL 2017	0.90	0.94	0.83
PL 2017	0.82	0.94	0.91
SP 2017	0.60	0.85	0.91
NL 2018	0.84	0.93	0.93
PL 2018	0.78	0.95	0.98
SP 2018	0.85	0.94	0.96

The high heritabilities reported in Table 1 are a good indication that the phenotypic variation was mainly due to genotypic variation and not field trends. Dry matter and tuber length had on average much higher heritabilities than tuber weight. This was not surprising as tuber weight is known to be a complex trait. The field trial in Spain of 2017 had the lowest heritabilities for tuber weight and tuber length. All other trials had on average, similar estimates of heritability. For more information on these traits, see Wilson *et al.* (2021).

#### Correlation between environments

Two components that indicate the presence of G×E is a lack of perfect genetic correlation as well as heterogeneity of genetic variance. Estimates of genetic correlations were extracted from the unstructured parametrization of the G×E model (equation (2)). In Table 2, the genetic correlations are shown for 2 traits, tuber weight, and tuber length.

**Table 2.** Genetic correlation between the 6 trials: above the diagonal are the correlations for tuber weight, while below the diagonals are the genetic correlations for tuber length.

Tuber weight					
**NL.17**	0.624	0.653	0.697	0.314	0.547
0.830	**PL.17**	0.799	0.685	0.422	0.637
0.708	0.873	**SP.17**	0.600	0.315	0.463
0.639	0.793	0.699	**NL.18**	0.302	0.695
0.643	0.835	0.803	0.872	**PL.18**	0.244
0.699	0.752	0.816	0.721	0.658	**SP.18**
Tuber length					

Characters in bold refer to the location and year: NL, PL, SP are Netherlands, Poland and Spain. NL.17 means Netherlands 2017.

**Fig. 4.**
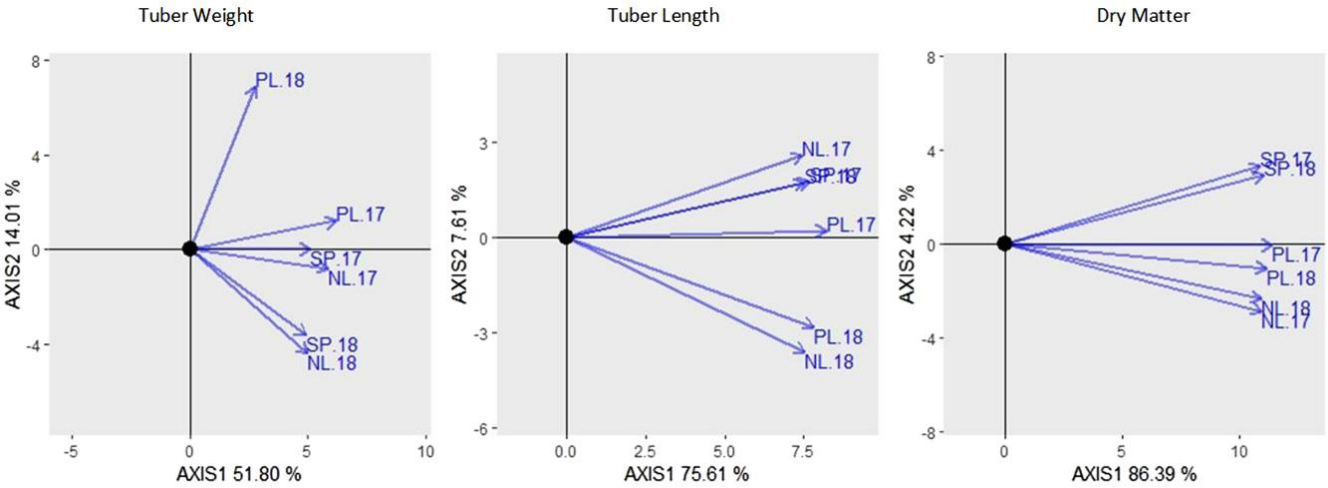
GGE plots for 3 traits across 6 environments, showing the extent of distinction between environments and therefore the amount of G×E potential.

From Table 2, we can see that tuber weight shows moderate to low genetic correlation, that vary noticeably across environmental pairs. This is evidence of G×E interactions, as genotype rankings are different across different environments. Tuber length had higher genetic correlations between environments than tuber weight, thus indicating less G×E interaction than tuber weight. The genetic correlations for dry matter (not shown) were quite high, ranging from 0.783 to 0.972 with an average of 0.877 indicating little to no presence of G×E interaction. Across the 3 traits, we see varying degrees of G×E, from quite low in dry matter to more substantial in tuber weight. The extent of G×E should also be considered in modeling strategies, as simpler G×E patterns can be captured by simpler models (compound symmetry) and more complex G×E patterns will require more complex models (unstructured). Looking at genetic correlations we can see that some environments are more similar for certain traits and this can be visualized with the GGE plot (Fig. 4).

The GGE plot gives an impression of the sources of environmental variation between trials: region or year. The 2017 trials seem to be quite similar across tuber weight and length suggesting that the difference in the year of planting was more influential than the difference in location. Dry matter is an exception where the locations of the trials are consistently more closely related than the years; this also occurs for tuber length in Spain. Tuber weight shows the most G×E, which is emphasized by the separation of the Poland 2018 trial, but not surprising when looking at the correlations in Table 2.

#### Model comparison

**Fig. 5.**
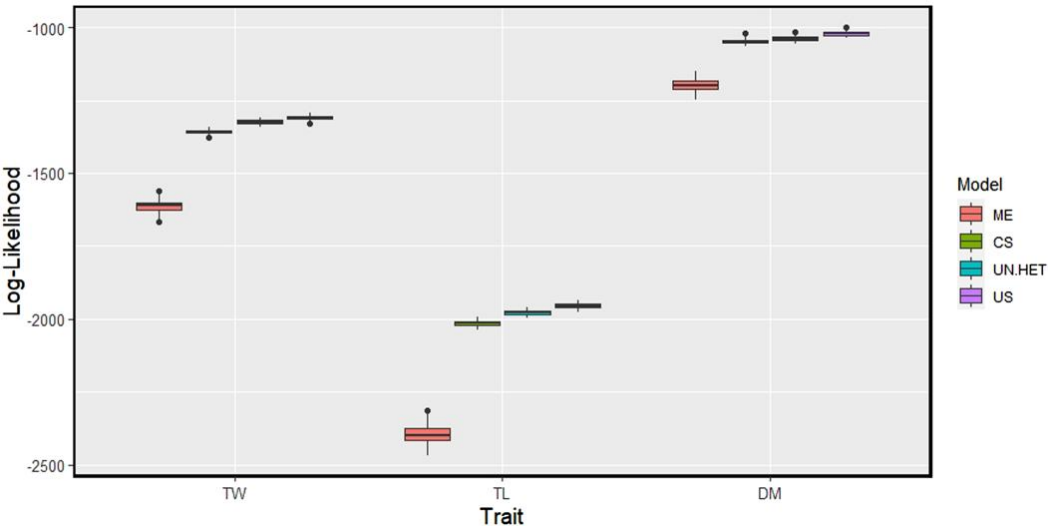
Comparison of model fit under scenario 2, using log-likelihood statistics. Traits analyzed: tuber weight (TW), tuber length (TL), and dry matter (DM). Models compared: main effects (ME), compound symmetry (CS), uniform Heterogeneity (UN.HET), unstructured (US).

Model comparison was done using scenario 2, where all 6 trials were used in the training set. Due to the variations in G×E patterns across the 3 traits, we expect that the best models for multienvironment prediction will vary from trait to trait. Using log-likelihood statistics, we can get a clearer picture of how well suited the model is for the data; a higher log-likelihood means a better fit. The log-likelihoods for the different multienvironment models and traits can be seen in Fig. 5.

For all traits, the inclusion of G×E interaction term fits the data better as seen by the higher log-likelihood scores in Fig. 5. The relative differences do vary across traits; the increase in likelihood when going from an ME model to a G×E model is less for dry matter than the other 2 traits. This indicates that modeling G×E is of greater importance in tuber length and tuber weight.

Based on Fig. 5, the more complex parametrizations of the G×E matrix, were better suited for all traits, even though the differences in likelihood between G×E models are small. This difference, however, is not consistent across traits. For tuber length and tuber weight, there is a noticeable difference between the compound symmetry model and the other 2 more complex models, which is not present in dry matter.

As more parameters are estimated, an increase in likelihood is expected; however, to test if the increase is significant a likelihood ratio test was performed. Across all traits, increasing model complexity sequentially gave significant improvements in explaining our data, as all *P*-values were below the common threshold of 0.05.

#### Scenario 1: Predicting tested genotypes for future years

**Fig. 6.**
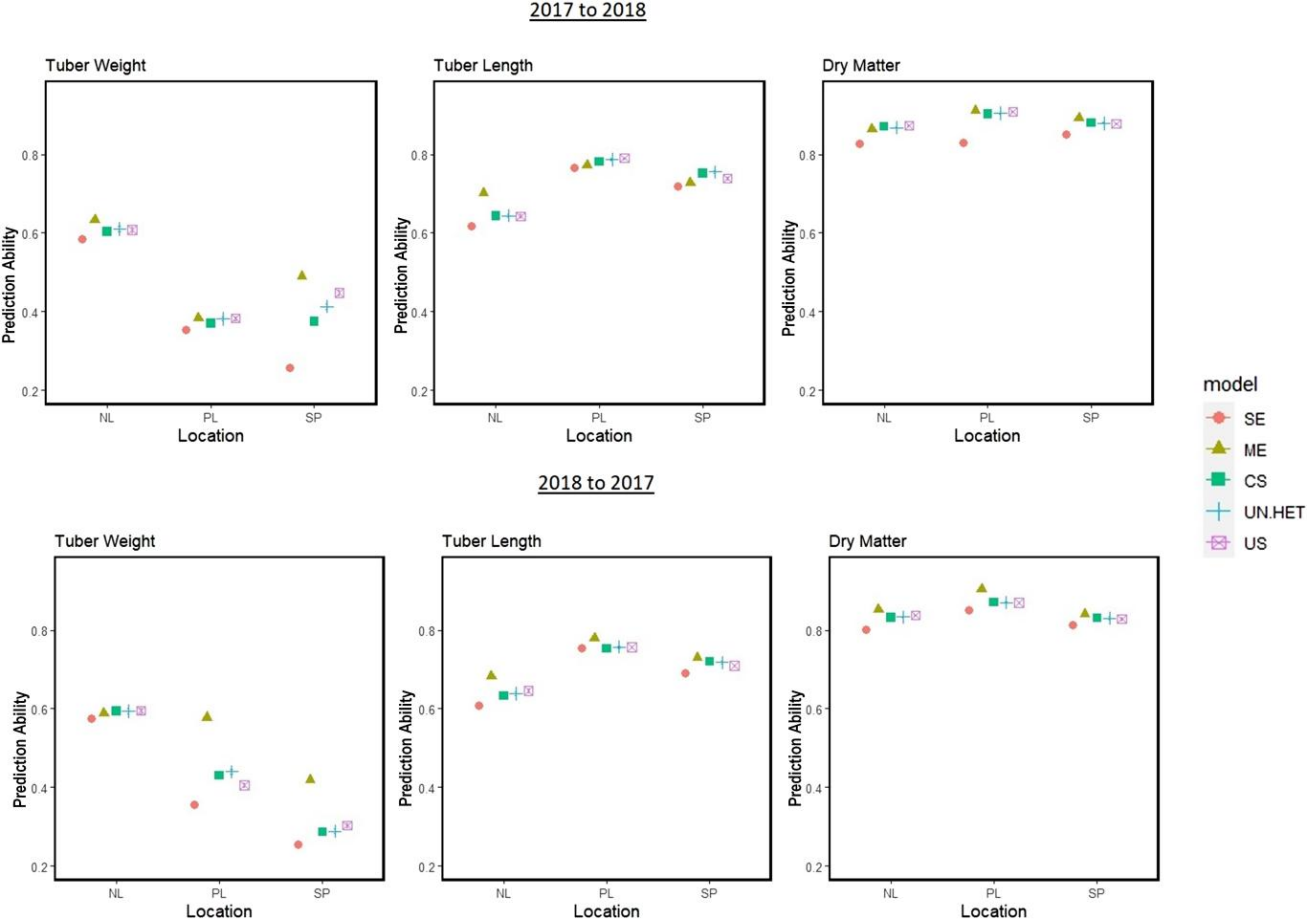
Results of scenario 1: Prediction abilities when predicting tested genotypes from one year to another. Regions are predicted from the same region in a different year (SE: single environment) or from all locations combined using multienvironment main effects model (ME), G×E compound symmetry (CS), G×E uniform heterogeneity (UN.HET), and G×E unstructured (US).

Using the 2017 and 2018 data, predictions were made from one year to the next in a particular region (Netherlands, Spain, or Portugal). The advantage of using multiple regions or just the region in question was assessed and the results can be seen in Fig. 6.

Following the order of heritabilities, dry matter was predicted the most accurate, followed by tuber length and finally tuber weight. The direction of prediction, meaning from 2017 to 2018 or vice versa, had no noticeable effect on prediction ability. Dry matter was predicted equally well in every region, while tuber weight was predicted substantially better in the Netherlands in comparison to the other 2 regions. As a result, prediction abilities for tuber weight in Spain and Poland benefited from the inclusion of the Netherlands’ trials. The same can be seen in tuber length, where the Dutch trials benefited from the information available from the other regions, where prediction abilities were higher for this trait.

Overall, multienvironment models that combined information across regions gave higher prediction abilities than predicting with a single environment model with data from just the target region. This was consistent across all regions and traits. Among the multienvironment models, the main genotypic effects model gave the most noticeable improvement in prediction ability for almost all trials. Modeling G×E did not improve prediction ability except for predicting tuber length in Poland and Spain. Focusing on the G×E models only, we see the patterns that were foreseen from the genetic correlations: tuber length and tuber weight have more complex G×E and therefore unstructured and uniform heterogeneity models did better than the simpler compound symmetry model in some trials. For dry matter, there was no difference among the G×E models and the compound symmetry parametrization was sufficient.

#### Scenario 2: Predicting untested genotypes in known conditions

**Fig. 7.**
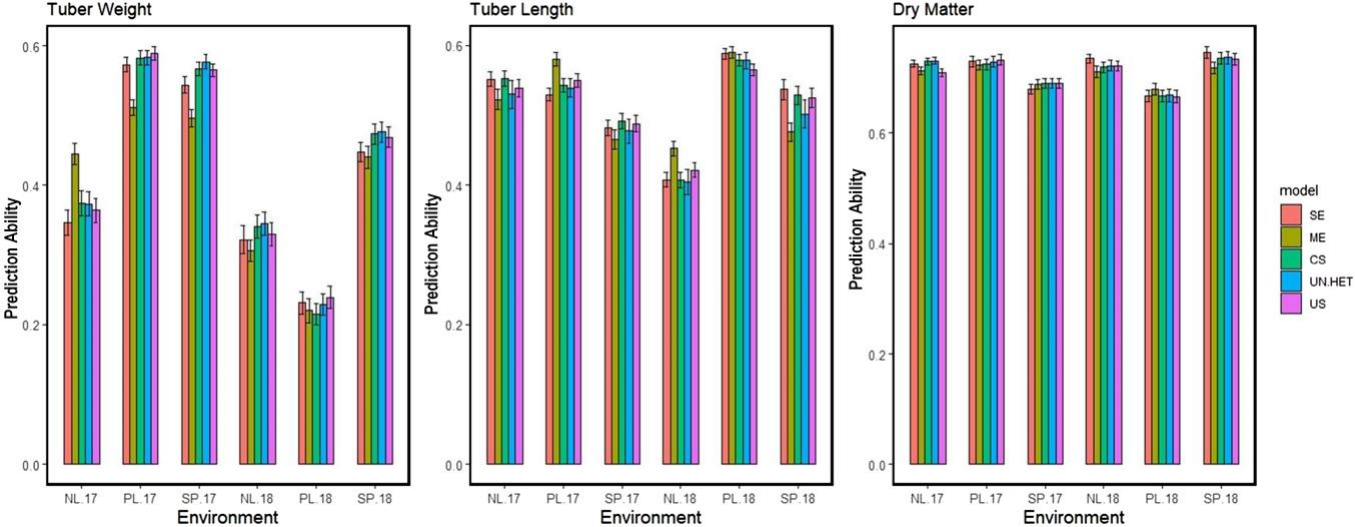
Scenario 2: Prediction abilities for across all 6 trials with various models: single environment (SE), main effects (ME), compound symmetry (CS), uniform heterogeneity (UN.HET), unstructured (US).

Treating all 6 trials as a separate environmental condition, we compared various models based on prediction ability. This comparison was done using scenario 2 (Fig. 2), where all environments are in the training set, and none of the genotypes to be predicted are used to train the model. The results can be seen in Fig. 7.

The first impression of the results in Fig. 7 further confirm the expectations for G×E, that were based on the correlations seen in Table 2. Results for dry matter, which had high correlations, show that there is very little impact of including G×E for multienvironment predictions, while tuber weight has improvements in prediction ability when modeling G×E. In the 1 trial where G×E improved prediction ability for dry matter (NL.17), compound symmetry was sufficient to capture the interaction effects. With such a high heritability and high genetic correlations between trials, predicting dry matter for new genotypes in known conditions can just as accurately be done by a single environment model.

The opposite can be said for tuber weight, where combining information across multiple trials always led to better prediction ability. With the exception of 1 trial (NL.17), those multienvironment models that include a term for G×E interaction gave better predictions. Among the remaining 5 trials, G×E models with more complex parametrizations (uniform heterogeneity and unstructured) were superior to the more simpler compound symmetry model.

Tuber length showed less of a clear trend; for most cases single environment prediction was just as good as or even more accurate than multienvironment models. Those multienvironment models that did improve prediction ability did so with only an ME model and no G×E interaction. Comparing prediction abilities of only the G×E models for this trait, we do not notice any pattern, as for some trials the simpler compound symmetry model is more effective, while for others the unstructured model is better; however, this comparison is not meaningful as the highest prediction abilities were already obtained without including G×E interactions.

#### Tuber weight (scenario 2)

Comparing the genetic correlations and heritabilities for tuber weight, we notice that some trials were quite different. Poland 2018 showed low genetic correlation with the other trials (Table 2 and Fig. 4), while Spain 2017 had a low heritability in comparison to the other trials (Table 1). These trials may be a potential source of noise to the multienvironment prediction model. Therefore, to test the impact of these trials, we looked at the prediction abilities using all trials, and compared it to the prediction abilities after removing Poland 2018 only and Spain 2017 only (Table 3).

**Table 3.** Prediction abilities in scenario 2 for tuber weight when using all trials, removing Poland 2018 only, and removing Spain 2017 only, for all multienvironment models and traits.

Models	Env. used	Predicted environments					
		NL.17	NL.18	PL.17	PL.18	SP.17	SP.18
	All	0.445	0.306	0.511	0.220	0.496	0.440
ME	No PL.18	0.422	0.281	0.480	—	0.460	0.434
	No SP.17	0.430	0.295	0.482	0.195	—	0.434
	All	0.374	0.341	0.582	0.215	0.567	0.473
CS	No PL.18	0.372	0.337	0.580	—	0.560	0.473
	No SP.17	0.370	0.340	0.579	0.207	—	0.471
	All	0.373	0.345	0.583	0.229	0.577	0.476
UN.HET	No PL.18	0.371	0.339	0.578	—	0.564	0.476
	No SP.17	0.370	0.345	0.578	0.224	—	0.475
	All	0.364	0.330	0.589	0.239	0.565	0.469
US	No PL.18	0.359	0.332	0.585	—	0.559	0.469
	No SP.17	0.364	0.331	0.575	0.241	—	0.472

For all models and environments, using all 6 environments gave higher prediction abilities than models that used 5 environments. For the ME model, it is clear that the information from these environments were useful and increased prediction ability but for the G×E models these differences were mostly negligible. Similarly, removing both these environments together did not improve prediction ability (results not shown).

**Fig. 8.**
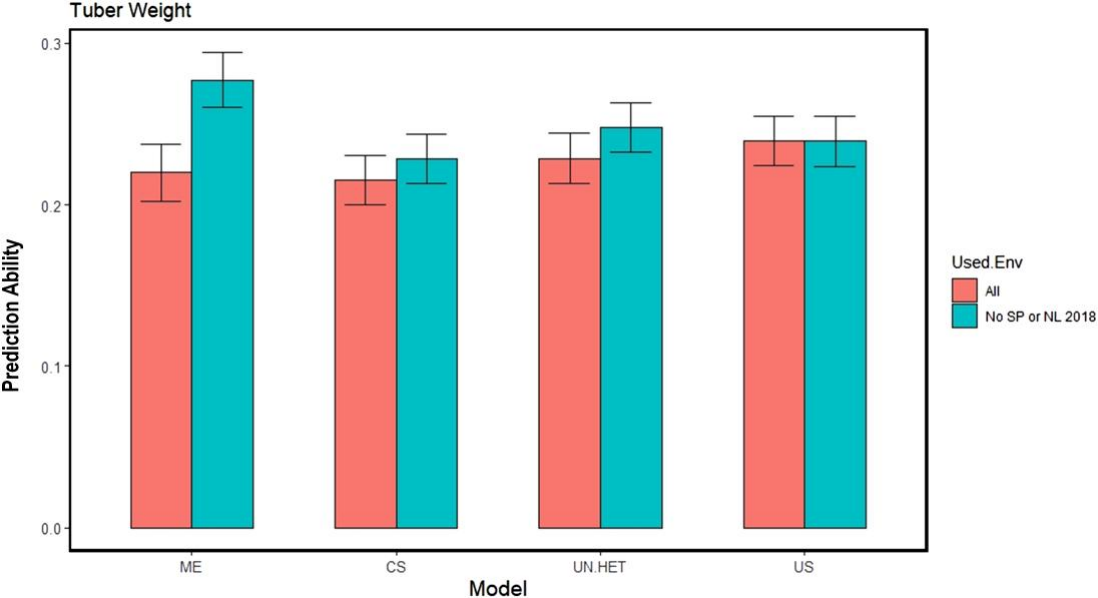
Prediction abilities in scenario 2 for Poland 2018 using all 6 trials vs prediction abilities when Spain and Netherlands 2018 are removed. Models compared: main effects (ME), compound symmetry (CS), uniform Heterogeneity (UN.HET), and unstructured (US).

To reverse perspectives, we look at prediction in Poland 2018 and how it may be affected by trials used in the prediction model. Referring to the estimated genetic correlations in Table 2 and the GGE biplot in Fig. 4, the 2018 trials in Netherlands and Spain are quite distant from the Poland trial of that year. Recall that in scenario 2 we are predicting new genotypes in 1 particular trial, using a model trained by the information from all 6 trials. For predicting tuber weight in the Poland 2018 trial, there are 2 trials that both have a low genetic correlation with Poland 2018. To have a closer look at how this impacts prediction ability, a similar comparison is done as seen previously, where predictions are made in Poland 2018 using all trials vs predictions made after both Spain and Netherlands 2018 are removed (Fig. 8).

Here, we see a different result from Table 3. The information added from Netherlands and Spain 2018 was not beneficial for making predictions in Poland 2018 and therefore prediction ability increased when these 2 environments were removed. This was clearest with the ME model. To a lesser degree, the models that included G×E interactions also benefited from removing these 2 environments, except for the unstructured model. (While the other 2 G×E models try to generalize the G×E patterns across environments, the unstructured model allows for unique correlations between all environments, and therefore was not affected when “noisy” environments are included.)

#### Scenario 3: Predicting untested genotypes for future years

**Fig. 9.**
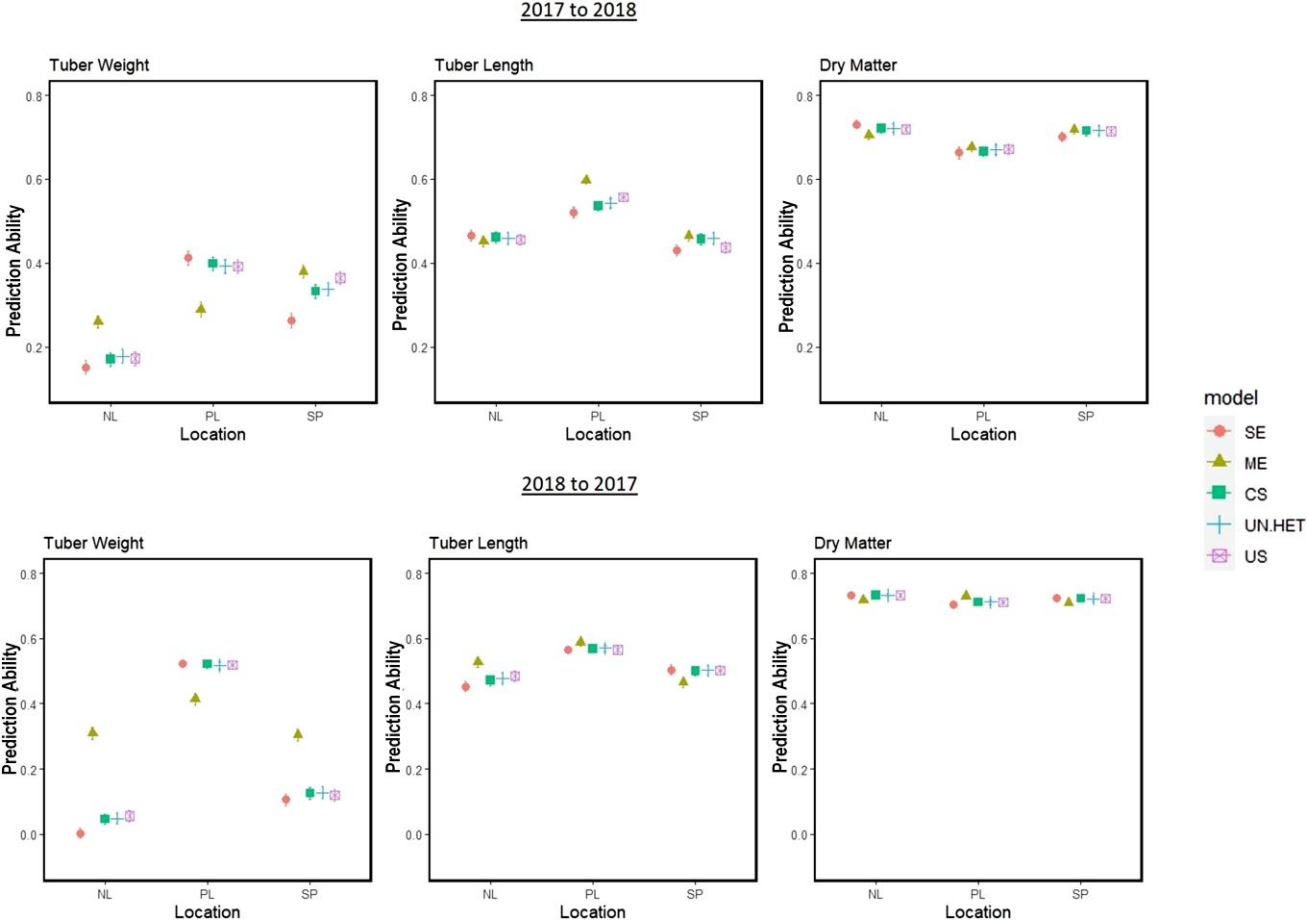
Results from scenario 3: Prediction abilities when predicting untested genotypes from one year to another. Regions are predicted from the same region in a different year (SE: single environment) or from all regions combined using multienvironment main-effect model (ME), G×E compound symmetry (CS), G×E uniform heterogeneity (UN.HET), and G×E unstructured (US).

In this scenario, we predicted the performance of new, untested genotypes from one year to the next in a particular region. With less information on genotype and environmental conditions available for combining, the impact of using multiple regions or just the location in question was compared (Fig. 9).

Similar to scenario 1, the ordering of traits in terms of prediction ability followed the ranking of their heritabilities. The direction of prediction did have an impact for tuber weight, as prediction abilities going from 2017 to 2018 were higher than prediction abilities going from 2018 to 2017 in 2 of the 3 regions, while in Poland this trend reversed. As expected, prediction abilities were noticeably lower than those calculated for scenario 1, as there is no information on genotype performance for cultivars in the predicted set.

From Fig. 9, we see that the combining of information across multiple regions did not always lead to more accurate prediction of new genotypes, when compared to predictions within a region. For tuber weight in Poland, and dry matter in the Netherlands, the single-region model gave prediction abilities better, or as good as the models that combined information from multiple regions. The only cases where multiregion prediction performed noticeably better than the single-region model were for tuber weight in Netherlands and Spain, and tuber length in Poland going from 2017 to 2018, and Netherlands from 2018 to 2017.

Listed above are the instances where combining the data across regions resulted in a substantial positive change, and these all occurred when the main-genotypic effect model was utilized. However, there are a few cases where the main-genotypic effects model caused prediction abilities to fall below the single-region model; namely tuber weight in Poland, and predicting tuber length in 2017 from 2018 data in the Spanish region. Among the G×E models, we see the same trends as in the other scenarios: dry matter shows no difference between the G×E parametrizations, while tuber length and tuber weight show evidence that the more complex G×E parametrizations are better suited than the simpler compound symmetry model.

The effectiveness of predicting from one year to a next depends largely on the trait and understanding the relationship between regions. This relation between trials (regions and years), can be seen from the GGE plot (Fig. 4). From the GGE plot, dry matter showed stronger genetic correlations between the same region of the trial than the same year of the trial; genetic correlation between the 2 Dutch trials (Spanish or Polish) were higher than the correlations from the same year. The prediction abilities for this trait show that the borrowing of information from multiple regions only added noise to the model and often did not improve predictions from year to year within a single region.

## Discussion

Including G×E in GP has been investigated in many crops including maize (Windhausen *et al.* 2012; Malosetti *et al.* 2013), barley (Malosetti *et al.* 2016), ryegrass (Fois *et al.* 2021), and coffee (Ferrão *et al.* 2019), to name just a few. Due to its ploidy and heterozygosity, G×E interactions in tetraploid potato has been theorized to be substantial (Jansky and Spooner 2018). In 1 recent study, modeling of G×E for GP has been shown to improve prediction ability of tuber weight (Ortiz *et al.* 2022). In the study by Ortiz *et al.*, predictions were made on tested genotypes (genotypes included in the training set as seen in scenario 1) and the different sites were all within 1 country in Europe. In this study, we conducted trials in different countries in Europe and investigated the G×E patterns for other traits, different breeding scenarios, and how that may affect modeling strategies and the trials included to train the model. Breeding programs that are in the later stages are interested in forecasting the future performance of existing material, in an effort to identify stable varieties. For this stage of breeding, the results of scenario 1 are more applicable. Scenario 2 is more applicable to breeding programs that are in the earlier stages and interested in testing new genotypes in known environments, in an effort to develop varieties well suited for a particular set of environmental conditions.

Overall, the impact of G×E interactions on prediction ability was quite low. More G×E was expected as trials were conducted in 3 distinctly different regions of Europe; however, this was not the case. All trials were closely monitored and subject to precise field management, which reduced the amount of G×E. This is good news for breeders as they can better control and forecast the performance of known cultivars in target regions, with the use of consistent management practices. There were limitations to this study due to the number of cultivars and trials, which are small in comparison to other multienvironment GP studies of other crops. Also, to understand the physiological mechanisms of G×E in potato, different conditions should be intentionally created, which was not the case in this study. Lastly, the population structure present in this diversity panel may not represent the population structure in breeding programs, and therefore affect the generalisability of the findings.

### Modeling G×E according to trait

We have seen that multienvironment GP of potato can improve prediction ability, across different scenarios. In scenario 2, where we had 6 trials in the training set, we had a closer look at how the estimated variance–covariance matrix influences model parametrization choices. Out of all the traits analyzed, only tuber weight showed evidence of significant G×E, which corresponds with the findings of Ortiz *et al.* (2022). Tuber length and dry matter showed little to no G×E which was evident from the genetic correlations reported in Table 2, and confirmed with the prediction abilities attained from different models.

The heritability estimates (Table 1) give an indication of heterogeneity of genetic variances, while Table 2 shows the heterogeneity of genetic correlations. Comparing these tables with the G×E covariance parametrizations, we can understand why certain models gave better predictions than others. The compound symmetry model assumes that the genetic variation is the same in each environment. Based on the heritability estimates in Table 1, this is a reasonable assumption for dry matter and tuber length, as all the heritabilities were quite similar. The uniform heterogeneous model assumes a unique genetic covariance for each environment, but a constant genetic correlation between environments. Based on the genetic correlations, we see that this can be applicable to dry matter and tuber length, however, not tuber weight. Prediction ability of tuber weight benefited from the more complex unstructured model which allowed correlations and variances to be environment specific, aligning with the heritability estimates and genetic correlations.

Modeling decisions are often made before predictions are carried out; assessment of models based on prediction ability in the test set, may lead to different conclusions, vs assessment of models based on likelihood statistics, that measure goodness-of-fit of the training data. Within the application of scenario 2, likelihood statistics were observed and used to compare models using the likelihood scores (Fig. 5) and likelihood ratio tests (not shown). As mentioned previously, for all traits, the more unstructured G×E model was significantly better than the simpler models. Likelihood scores were used to help identify the most suitable parametrization for the variance–covariance matrix, but such a model as assessed on the training set does not automatically transfer to the test set. Also we must consider that for the likelihood ratio tests, not many parameters were estimated in this study. For scenario 2, we look at 6 trials, therefore the number of parameters estimated for variances and covariances in the unstructured model are relatively small in comparison to many breeding programs.

### Predicting from year to year

In many G×E studies, the potential of using years of past data to predict forward is of keen interest. In this study, we were not able to use years of data to predict forward; however, we did get an impression of using one year to predict another, and the results were encouraging from a breeder’s perspective. Within the same region, we are able to predict from one year to the other, and in the majority of cases, this improved when information was combined from multiple regions. The added value of incorporating G×E in the multienvironment prediction model was small in cases with high G×E, while in cases with low G×E it did not help. For each region, there is only 1 year of information; however, there are some valuable concepts from the scenarios explored in this study.

#### Scenario 1

In this scenario, we made year to year predictions of tested genotypes, i.e. genotypes included in the training set (Fig. 6). We clearly see that multiregion prediction models performs consistently better than single-region models, when forecasting the performance of genotypes in the upcoming year. The improvement in prediction abilities were the greatest with the main-genotype effect model and not models that included a G×E interaction term. It is true that among the G×E models we see differences in model performance depending on the genetic correlations of the traits, as discussed previously in the section *Modeling G×E according to trait*; however, in this section, we observe another result. Because predictions are made on genotpyes that occur in one of the regions included in the training set, therefore adequately estimating the genotpyic main effect, this main-effect model benefits the most and seems to be a fitting choice for this scenario. It is especially helpful when prediction ability in a trial of one of the training regions is higher than ability in the region where predictions are to be made. This shows the added value of including trials in very different regions of Europe for forecasting.

#### Scenario 3

In this scenario, we made year to year predictions of untested genotypes, i.e. genotypes not included in the training set (Fig. 9). As expected, the prediction abilities were better in scenario 1 where we predicted tested genotypes, as also seen in other studies (Burgueño *et al.* 2012; Malosetti *et al.* 2016). In this scenario, we notice that there are many cases where combining regions lead to higher prediction ability, but we also see cases where the multiregion model can perform worse than the single-region model. When this occurred, it was the main-genotypic effect model being applied. The absence of the information on genotype makes the main-genotype effect model a riskier choice, a pattern also observed in scenario 2 (Fig. 7). When combining information in scenarios with untested genotypes, the G×E models perform as well as and sometimes better than the single-region model. Unlike the main-genotype effect model, the G×E models never substantially reduced prediction ability.

For dry matter in particular, looking at the genetic correlations and the GGE biplot (Fig. 4), genotypic performance appears to be more consistent according to region than year. For that reason a model within that single region was enough to predict untested genotypes from one year to the other. For example, for predicting new genotypes in Spain 2018, better prediction ability was attained when using Spain 2017 only and not all three 2017 trials together. This introduces the discussion topic about adding multiple “distant” trials to the training set (in terms of genetic correlation), and its impact on prediction ability.

### Tuber weight and the training set of trials (scenario 2)

In scenario 2, we had 6 trials or trial conditions, and we were predicting new untested genotypes for these a known trial condition. This allowed us to investigate the impact of the training set of environments, or in this case, the training set of trial conditions. When applying multitrial prediction to tuber weight, we see that the set of trials used for training is important (Fig. 8). The Poland 2018 trial had low correlation with all other trials so it did not benefit from combining information across trials. However, when the 2 trials that had the lowest correlations with Poland 2018 were removed, prediction ability improved. This result shows that we cannot simply utilize all trials available when applying multitrial GP, and must instead, investigate the genetic correlation between the target trial condition, and the trials that are to potentially be used to train the model. Of course it is curious that removing Poland 2018 to make predictions in the other trials did not improve prediction ability. A possible explanation is that 1 singular trial with low genetic correlation, and not multiple trials with low genetic correlation, may not contribute enough noise to negatively impact prediction ability. The advice to not use a multienvironment main-genotypic effect model when predicting untested genotypes also gets support from the result in Poland 2018 (Fig. 8). We notice that this model benefits the most from removing the lowly correlated trials; however, it can be viewed inversely as the model that has the highest potential to go wrong when the composition of trials in the training set is not suitable.

The trial with the lowest heritability benefited the most from including trials with high heritabilities (Spain 2017). Inversely, it was expected that removing this trial to make predictions where the heritabilities were higher, would improve prediction ability. This was not observed in this study, possibly due to the same reason as above, 1 trial was not enough to negatively impact prediction ability. Another explanation is that even though the trial has a lower genetic signal, it is still extra information that has a decent correlation with the target trial, and therefore useful.

## Conclusions

- For multienvironment GP, it is important to consider the prediction scenario when choosing a modeling strategy.
- For tested genotypes (scenario 1), the multiregion main-genotypic effect model was the most suitable choice for combing information across regions. For untested genotypes (scenarios 2 and 3), the same ME model can negatively impact prediction ability and models that include G×E interactions were more reliable.
- Predicting a target set of environment conditions can be negatively impacted when multiple trials that have low genetic correlation are included in the training of the model.
- Genotype by environment interactions are to be expected from multiregion trials; however, they can be minimized with consistent management practices.

## Supplementary Material

jkae011_Supplementary_Data

## Data Availability

The data used in this study are available in the Supplementary Material. Supplementary File S1 contains phenotypic information including BLUEs, as well as the location and year of the trial. Supplementary File S2 contains marker information. Supplemental material is available at G3 online.
